# SETD6 mediates selective interaction and genomic occupancy of BRD4 and MITF in melanoma cells

**DOI:** 10.1093/narcan/zcaf023

**Published:** 2025-08-07

**Authors:** Tzofit Elbaz Biton, Michal Feldman, Tomer Davidy, Nili Tickotsky Moskovitz, Liron Levin, Daniel Sevilla, Colin R Goding, Emily Bernstein, Dan Levy

**Affiliations:** The Shraga Segal Department of Microbiology, Immunology and Genetics, Ben-Gurion University of the Negev, P.O.B. 653, Be’er-Sheva 84105, Israel; National Institute for Biotechnology in the Negev, Ben-Gurion University of the Negev, P.O.B. 653, Be’er-Sheva 84105, Israel; The Shraga Segal Department of Microbiology, Immunology and Genetics, Ben-Gurion University of the Negev, P.O.B. 653, Be’er-Sheva 84105, Israel; National Institute for Biotechnology in the Negev, Ben-Gurion University of the Negev, P.O.B. 653, Be’er-Sheva 84105, Israel; The Shraga Segal Department of Microbiology, Immunology and Genetics, Ben-Gurion University of the Negev, P.O.B. 653, Be’er-Sheva 84105, Israel; National Institute for Biotechnology in the Negev, Ben-Gurion University of the Negev, P.O.B. 653, Be’er-Sheva 84105, Israel; Bioinformatics Core Facility, Ilse Katz Institute for Nanoscale Science and Technology, Ben-Gurion University of the Negev, P.O.B. 653, Be’er-Sheva 84105, Israel; Bioinformatics Core Facility, Ilse Katz Institute for Nanoscale Science and Technology, Ben-Gurion University of the Negev, P.O.B. 653, Be’er-Sheva 84105, Israel; The Research Support Laboratories of Ilse Katz Institute for Nano-Science and Technology, Ben-Gurion University of the Negev, P.O.B. 653, Be’er-Sheva 84105, Israel; Ludwig Institute for Cancer Research, Nuffield Department of Clinical Medicine, University of Oxford, Headington, Oxford OX3 7DQ, United Kingdom; Department of Oncological Sciences, Tisch Cancer Institute, Icahn School of Medicine at Mount Sinai, New York, NY 10029, United States; The Shraga Segal Department of Microbiology, Immunology and Genetics, Ben-Gurion University of the Negev, P.O.B. 653, Be’er-Sheva 84105, Israel; National Institute for Biotechnology in the Negev, Ben-Gurion University of the Negev, P.O.B. 653, Be’er-Sheva 84105, Israel

## Abstract

Aberrant transcriptional programs mediate malignant transformation of melanoma, the most aggressive form of skin cancer. The lysine methyltransferase SETD6 has been implicated in regulating transcription, cell adhesion, migration, and other processes in various cancers; however its role in melanoma remains unexplored. We recently reported that SETD6 monomethylates the BRD4 at K99 to selectively regulate transcription of genes involved in mRNA (messenger RNA) translation. Here, we observed that BRD4 methylation at K99 by SETD6 occurs in melanoma cells. Knockout of SETD6 or a point mutation at BRD4-K99 disrupts BRD4 genomic occupancy. In addition, we show that SETD6 interacts with MITF, a master transcription factor in melanocytes and melanoma, and influences the genomic distribution of MITF. Mechanistically, we uncover a novel chromatin-localized interaction between BRD4 and MITF in melanoma. Our data suggest that BRD4 binds MITF in melanoma cells and that this interaction is dependent on both SETD6-mediated methylation of BRD4 and MITF acetylation. This chromatin complex plays a pivotal role in selective recruitment of BRD4 and MITF to different genomic loci in melanoma cells.

## Graphical Abstract

**Graphical Abstract:**
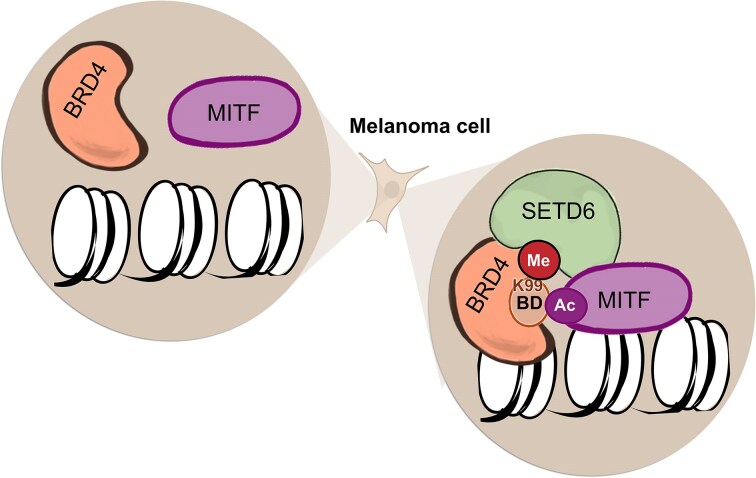

## Introduction

Melanoma, a neoplasm of melanocytic origin, is the most severe human skin cancer [1]. Malignant transformation in melanoma is caused by mutations in genes that are responsible for cell proliferation and apoptosis, epigenetic changes, changes of adhesion ability, or production of autocrine growth factors. These aspects disturb the signal transduction pathways in melanocytes, the skin cells producing the pigment melanin [2]. Recent studies have revealed a complex involvement of epigenetic mechanisms in the regulation of transcriptional programs in melanoma, including methylation, chromatin modification and remodeling, and the diverse activities of noncoding RNAs and transcription factor (TF) activity [3]. The microphthalmia-associated transcription factor (MITF) is a master regulator of melanocyte development and controls many aspects of melanocyte and melanoma biology, including the cell cycle, metabolism, DNA damage repair, survival, differentiation, and proliferation [4]. Further, MITF is amplified in a fraction of human melanomas [1] and has been termed a lineage survival oncogene [5].

The transcription regulator BRD4 is a member of the bromodomain (BD) and extra-terminal domain (BET) protein family. BRD4 contains two conserved BDs that specifically recognize acetylated lysine residues on histone and non-histone proteins [6, 7]. In recent years, many studies have demonstrated a notable therapeutic potential of BET inhibitors, which target BRD family members and displace them from chromatin [8, 9]. In melanoma, BRD4 is overexpressed and essential for tumor growth *in vivo* [10] and interacts with MITF to regulate the expression of genes important for melanin synthesis in melanocytes [11]. Given this role of BRD4 in melanocyte biology, we hypothesized that SETD6-mediated methylation of BRD4 would modulate the transcriptional program in melanoma cells.

Post-translational modifications (PTMs) contribute to changing protein properties, transducing cellular signals, and regulating protein–protein interactions, thus serving as an important mechanism for the regulation of many signaling pathways and biological processes [12]. Several studies have demonstrated the importance of lysine methylation for regulating epigenetic processes and fundamental cellular signaling pathways via methylation of non-histone proteins [13–15]. As part of its structural characteristics, lysine can be mono-, di-, or trimethylated, whereby *S*-adenosyl-l-methionine (SAM/AdoMet) serves as the methyl donor for this PTM. Lysine methylation is a dynamic modification that is written by protein lysine methyltransferases and removed by lysine demethylases [16]. There are ∼50 members of the PKMT family, of which most contain a conserved Su(var), enhancer of zeste, trithorax (SET) domain, which is responsible for the enzymatic activity [17]. SET domain-containing protein 6 (SETD6) is a monomethyltransferase containing the catalytic SET domain and a Rubisco substrate-binding domain that mediates protein–protein interactions [17, 18]. The enzymatic activity of SETD6 was shown to be involved in the regulation of diverse processes including transcription, cell adhesion, and migration, among several others [17–29]. However, the role of SETD6 in melanoma and the pathways through which it might act remain unclear. We recently showed that SETD6 functions as a molecular switch that methylates chromatin-bound BRD4 at lysine-99 (K99). Consequently, BRD4 methylation affects the recruitment of the E2F1 TF to genes involved in mRNA translation in breast cancer cells [28].

Here, we show that SETD6 binds and methylates BRD4 at K99 in melanoma cells to modulate the genomic distribution of BRD4 and MITF, and that BRD4 binds to MITF *in vitro* and in cells. Mechanistically, our data suggest that the interaction between BRD4 and MITF is SETD6-mediated and BRD4 methylation-dependent and requires acetylation of MITF. We propose that the formation of this complex at chromatin has a role in transcription regulation of genes that are important for melanoma initiation and progression.

## Materials and methods

### Plasmids

All BRD4 plasmids were previously described [28]. pcDNA-FLAG BRD4-N140A and pcDNA-FLAG BRD4-N140F were generated using site-directed mutagenesis. Primer sequences are shown in Supplementary Table S1. SETD6 plasmids were also produced in previous papers [19, 21–23, 26–30]. Human MITF-A coding sequence was excised from plasmid pEGFP-N1-MITF-A (Addgene #38132). Human MITF-M coding sequence was excised from plasmid pCMV-Tag4A-MITF-M, which was kindly provided by the laboratory of Prof. Victoria P. Belancio. MITF coding sequences were amplified by using polymerase chain reaction (PCR) with compatible primers as indicated in Supplementary Table S1. Amplified PCR products were digested with AscI and PacI restriction enzymes and subcloned into pcDNA3.1 3× Flag and pcDNA3.1 3× HA plasmids, as well as pET-Duet-His plasmid for protein purification. KAPA HiFi HotStrart ReadyMix (KAPA Biosystems) was used for the PCR reactions. All cloned plasmids were confirmed by sequencing.

### Cell line treatment

Melanoma cell lines SKmel147 (NRAS mutant) and human embryonic kidney cells (HEK293T) were maintained in Dulbecco’s modified Eagle’s medium (Sigma, D5671) with 10% fetal bovine serum (Gibco, 10270106), 1% penicillin–streptomycin (Sigma, P0781), 2 mg/ml l-glutamine (Sigma, G7513), and nonessential amino acids (Sigma, M7145). Cells were cultured at 37°C in a humidified incubator with 5% CO2.

### Transfection and infection

Transfections of HEK293T were performed using polyethyleneimine (PEI) reagent (polyethyleneimine Inc., 23966) and Mirus reagent. TransIT-X2 (MC-MIR-6000-1.5) was used for melanoma cells (SKmel147), according to the manufacturer’s instructions.

For stable transfection in SKmel147 cell lines, retroviruses were produced by transfecting HEK293T cells with the indicated pWZL constructs (empty, Flag BRD4 wild type 1–477 aa or Flag BRD4 K99R 1–477 aa) and with plasmids encoding VSV and gag-pol. Target cells were infected with the viral supernatants and selected with 250 μg/ml hygromycin B (TOKU-E), respectively.

### SETD6 knockout by CRISPR/Cas9

For SKmel147 CRISPR/Cas9 SETD6 knockout (KO) cells, four different gRNAs for SETD6 (Supplementary Table S1) were cloned into lentiCRISPR plasmid (Addgene, #49535). 3 × 10^5^ melanoma cells per six-well plate were plated and transfected with Mirus reagent, TransIT-X2, according to the manufacturer’s protocol. Following transfection and puromycin selection (2.5 μg/ml), single clones were isolated, expanded, and validated by sequencing.

### RNA-sequencing analysis

Total RNA was extracted from SKmel147 cells (SETD6 control versus KO) using the NucleoSpin RNA Kit (Macherey-Nagel). Samples were prepared in two biological replicates for control cells and four biological replicates for SETD6 KO cells. Libraries were prepared using the INCPM-mRNA-seq protocol. Briefly, the polyA fraction (mRNA) was purified from 500 ng of total input RNA, followed by fragmentation and the generation of double-stranded complementary DNA. Afterward, an Agencourt Ampure XP beads cleanup (Beckman Coulter), end repair, A base addition, adapter ligation, and PCR amplification steps were performed. Libraries were quantified by Qubit (Thermo Fisher Scientific) and TapeStation (Agilent). Sequencing was done on a Hiseq instrument (Illumina) using two lanes of an SR60_V4 kit, allocating 20M reads per sample (single read sequencing).

### Data processing of SETD6 KO RNA-seq

The analysis of the raw sequence reads was carried out using the NeatSeq-Flow platform (DOI: https://doi.org/10.1101/173005). The sequences were quality trimmed and filtered using Trim Galore (version 0.4.5) (quality cutoff = 25, length cutoff = 25) and cutadapt (version 1.15) (DOI: https://doi.org/10.14806/ej.17.1.200). Alignment of the reads to the human genome (GRCh38) was done with RSEM (version 1.2.28) [31] (option “bowtie2”) and calculation of number of reads per gene per sample was also done with RSEM. Quality assessment of the process was carried out using FASTQC (version 0.11.8) and MultiQC (version 1.0.dev0) [32]. Genes with low expression values (mean count <1 over all samples) were excluded from differential expression analysis.

Read counts for differential gene expression were analyzed with the DESeq2 R package [33] using the the NeatSeq-Flow platform DESeq2 module. For the four CRISPR-treated KO samples versus the two control samples, genes with fold change ≥1 and adjusted *P* < .05 were considered as significantly differentially expressed genes (DEGs). Significant genes were clustered using the “eclust” function from the factorextra R package (10.32614/CRAN.package.factoextra) with the default restriction of maximum clusters. Enrichment for Gene Ontology (GO) biological processes and KEGG pathways were performed using clusterProfiler v4.0 R package (DOI: 10.1016/j.xinn.2021.100141). RNA-seq data were deposited into the Gene Expression Omnibus database under accession number GSE298160.

### ChIP (Chromatin immunoprecipitation) enrichment analysis

ChIP-X enrichment analysis is a gene-set enrichment analysis tool tailored to test whether query gene sets are enriched with genes that are putative targets of TFs. ChIP enrichment analysis (ChEA) utilizes a gene-set library with TFs labeling sets of putative target genes curated from published ChIP-chip, ChIP-seq, ChIP-PET, and DamID experiments [34]. For SKmel147 SETD6 RNA-seq, DEGs (*P* < .05) were analyzed in the Enrichr platform for GO biological processes and Hallmark gene sets.

### Colony-formation (clonogenic) assay

Cells were seeded in six-well plates at 1000, 2000, or 5000 cells per well and incubated at 37°C incubator for 5–10 days. Cell colonies were washed twice with phosphate-buffered saline (PBS), fixed, and stained with 0.5% crystal violet in 20% methanol for 5 min. Representative wells were photographed. Each experiment was performed in duplicate (technical replicates). Crystal violet staining was solubilized in 2% sodium dodecyl sulfate (SDS) and quantified at 550 nm using a Tecan Infinite M200 plate reader.

### Adhesion assay

For cell adhesion assay, 3 × 10^5^ cells/well were plated on 96-well plate for 1 h, followed by a PBS wash and crystal violet staining (0.5% crystal violet in 20% methanol). The crystal violet stained cells were solubilized in 2% SDS and quantified at 550 nm using a Tecan Infinite M200 plate reader.

### Antibodies and western blot analysis

Primary antibodies used were anti-Flag (Sigma, F1804), anti-HA (Millipore, 05-904), anti-Actin (Abcam, ab3280), anti-SETD6 (Genetex, GTX629891), anti-pan-methyl (Cell Signaling, 14679), anti-pan-acetyl lysine (Cell Signaling, 9681s) anti-BRD4 (Bethyl, A700-004), anti-BRD4 K99me1 antibody (U292-FT) [28], anti-MITF (Santa Cruz, sc-515925), anti-histone 3 (H3) (Abcam, ab10799), and GFP (Abcam, ab290). HRP-conjugated secondary antibodies, goat anti-rabbit and goat anti-mouse, were purchased from Jackson ImmunoResearch (111-035-144 and 115-035-062, respectively). For western blot (WB) analysis, cells were homogenized and lysed in RIPA (radioimmunoprecipitation assay) buffer [50 mM Tris–HCl (pH 8), 150 mM NaCl, 1% Nonidet P-40, 0.5% sodium deoxycholate, 0.1% SDS, 1 mM dithiothreitol, and 1:100 protease inhibitor mixture (Sigma)] for 10 min on ice. Proteins were denatured with Laemmli sample buffer (250 mM Tris–HCl, pH 6.8, 10% SDS, 30% glycerol, 5% β-mercaptoethanol, a pinch of bromophenol blue), boiled for 5 min at 95°C, and were resolved on 8%–12% SDS–polyacrylamide gel electrophoresis (PAGE), followed by blotting onto PVDF membranes and analysis with appropriate antibodies.

### Chromatin extraction by biochemical fractionation and protein–protein interaction analysis by immunoprecipitations

Cells were cross-linked using 1% formaldehyde (Sigma) added directly to the medium and incubated on a shaking platform for 10 min at room temperature (RT). The cross-linking reaction was stopped by adding glycine to a final concentration of 0.125 M and incubating for an additional 5 min on the shaking platform. Cells were washed twice with PBS and then lysed in 1 ml cell lysis buffer (20 mM Tris–HCl, pH 8, 85 mM KCl, 0.5% Nonidet P-40, 1:100 protease inhibitor cocktail) for 10 min on ice. Nuclear pellets were resuspended in 120–200 μl nuclei lysis buffer (50 mM Tris–HCl, pH 8, 10 mM EDTA, 1% SDS, 1:100 protease inhibitor cocktail) for 10 min on ice and then sonicated (Bioruptor, Diagenode) at high power settings for three cycles, 6 min each (30 s on/off) or sonicated by focused-ultrasonicator (ME220, Covaris). Samples were centrifuged (20 000 × *g*, 15 min, 4°C) and the soluble chromatin fraction was collected.

For protein–protein interaction analysis with endogenous BRD4 or MITF, the soluble chromatin was precleared with Magna ChIP™ Protein A+G Magnetic Beads (Millipore, 16-663) for 1 h. Then, 3 or 6 μl of anti-BRD4/MITF antibody was added to the precleared sample for each IP reaction and the tubes were rotated in a shaker at 4°C overnight. Then, 20 μl Magna ChIP™ Protein A+G Magnetic Beads were added to each immunoprecipitation (IP) reaction, followed by incubation in a shaker at 4°C for 2 h. The tubes were then placed on a magnetic separator to capture magnetic beads and the associated proteins. The supernatants were removed and the magnetic beads were washed with 1 ml of 1× PBS. For pan-methyl IP, cell lysates in RIPA buffer were precleared with A/G agarose beads (Santa Cruz, SC-2003) for 1 h and then incubated overnight at 4°C with pan-methyl antibody that was preconjugated to A/G agarose beads. The immunoprecipitated complexes were washed once with PBS and then resolved in protein sample buffer and analyzed by WB. For SAHA (SuberoylAnilide Hydroxamic Acid) treatment, cells were treated for 4 h with 5–20 μM compound or with equivalent amount of dimethyl sulfoxide as a vehicle control. SAHA was provided by Dr D. Toiber (Ben-Gurion University, Israel). For JQ1 treatment, lysates of cells were treated with 1 μM JQ1 and incubated with primary antibody for IP. JQ1 was provided by Dr V. Shoshan-Barmatz (Ben-Gurion University, Israel).

### Recombinant protein purification

*Escherichia coli* BL21 transformed with a plasmid expressing a His-tagged protein of interest was grown in LB media. Bacteria were harvested by centrifugation after IPTG (Isopropyl β-d-1-thiogalactopyranoside) induction and lysed by sonication on ice (25% amplitude, 1 min total, 10/5 s on/off). Homogenization was performed with ice-cold lysis buffer containing PBS, 10 mM imidazole, 0.1% Triton X-100, and 1 mM PMSF. After adding 0.25 mg/ml lysozyme for 30 min, the lysates were subjected to sonication on ice (18% amplitude, 1 min total, and 10 s on/off). The tagged fusion proteins were purified on a His-Trap column using an AKTA Pure protein purification system (GE). The proteins were eluted with 0.5 M imidazole in PBS buffer, followed by overnight dialysis (PBS, 10% glycerol).

### In vitro methylation assay

The methylation assay reaction (total volume of 25 μl) contained 1 μg His-Sumo BRD4, 4 μg His-SETD6, 1 μl of 2mCi of 3H-labeled *S*-adenosyl-methionine (PerkinElmer, AdoMet), and PKMT buffer (20 mM Tris–HCl, pH 8, 10% glycerol, 20 mM KCl, 5 mM MgCl_2_). The reaction tubes were incubated overnight at 30°C. Then, the reactions were resolved by SDS–PAGE for Coomassie staining (Expedeon, InstantBlue) or autoradiography (Typhoon FLA 7000, GE).

### Enzyme-linked immunosorbent assay

Two micrograms of recombinant proteins [bovine serum albumin (BSA), MBP-RelA, His-Sumo BRD4, and His MITF] in PBS were added to a sticky surface (Greiner Microlon) 96-well plate and incubated for 1 h at RT, followed by 3% BSA blocking in 1× PBS and 0.1% Tween (PBST) for overnight incubation at RT. After three washes with PBST, 0.5 μg GST-SETD6 or GST protein (negative control) diluted in 1% BSA in PBST was added to the wells for 1 h at RT. Plates were washed and incubated with primary antibody (anti-GST, 1:4000 dilution), followed by incubation with an HRP-conjugated secondary antibody (goat anti-rabbit, 1:2000 dilution) for 1 h. Finally, TMB reagent and then 1N H_2_SO_4_ were added; the absorbance at 450 nm was detected using a Tecan Infinite M200 plate reader. Results are represented as relative absorbance compared to GST or BSA/His-Sumo.

### Cleavage under targets and release using nuclease

CUT&RUN (cleavage under targets and release using nuclease) was performed using the CUTANA™ Assay Kit (EpiCypher) according to the manufacturer’s instructions. An input of 1 × 10^6^ cells per sample was processed according to the manufacturer’s protocol. More specifically, with the use of nuclear extraction buffer, the CUT&RUN assay was performed directly on nuclei. Digitonin was used at a final concentration of 0.01% for nuclear permeabilization. 0.5 μg of control antibodies (IgG and H3K4me3) was used as recommended and 1 μg of anti-BRD4 (EpiCypher 13-2003), anti-MITF (Santa Cruz, sc-515925), and anti-Flag (Sigma, F1804) antibodies were used per sample. Libraries were quantified with the Qubit dsDNA HS Assay Kit and DNA fragment sizes were assessed using an Agilent TapeStation. Resulting libraries were sequenced with 50–150-bp paired-end reads, with 15M reads per sample using a NextSeq 2000 system from Illumina through the Center for Advance Genomics, IKI, BGU.

### CUT&RUN data analysis

The analysis was carried out using the NeatSeq-Flow platform [35] (https://doi.org/10.1101/173005). Raw sequencing data in fastq format were trimmed by Trim Galore v0.4.5 (length = 25, q- 25), reads that were too short were discarded. FastQC v0.12.0 was used for reads quality control. BWA Mapper (version 0.7.12, default parameters t = 20, B = 5) [36] was used to align paired-end reads to reference human genome assembly hg38 and to the spike-in control (*E. coli*) reference genome assembly. SAMtools [37] was used to remove PCR duplicates and create coordinate-sorted BAM files. Normalization between samples and IgG was performed with a custom script that calculates read scaling factor based on spike-in DNA reads of each sample. The scaling factor was then used in the bamCoverage v3.5.6 by deepTools [38] to normalize each sample according to its spike-in, Bigwig files were created by bamCoverage and reads mapped to blacklisted areas designated by ENCODE [39] were filtered. Reads that refer to off-chromosome locations were removed with BedClip v377 and sorted by Bedtools v2.30.0 [40]. Bigwig files were then created with bedGraphToBigWig by deepTools [38]. For BRD4, peaks were called by MACS (version 3.0.0) [41] (“callpeak” function, options “nomodel,” “broadpeak,” broad-cutoff = 0.05). For MITF, peaks were called by SEACR v1.3 (using the top 5 percentile as the high-confidence peak calibration in “stringent” mode) [42]. Peaks within 100 bp were then merged and sorted with Bedtools.

The union of peaks from each two replicates (BRD4, Flag-BRD4, and MITF) were then intersected by Bedtools (version 2.31.0) [40], resulting in a peak list that included only peaks that exist in both replicates. Peaks were viewed in IGV (version 2.16.1) [43] and annotated with ChIPseeker [44, 45]. Matrices were generated with DeepTools createMatrix, and Heatmaps were plotted with deepTools plotHeatmap [38]. CUT&RUN data were deposited into the Gene Expression Omnibus database under accession number GSE298161.

### Proximity ligation assay

Cells were cultivated on coated coverslips, washed with PBS, and fixed in cold 4% paraformaldehyde at RT for 15 min. Cell permeabilization was performed using 0.5% Triton X-100 in PBS for 10 min at RT. Proximity ligation assay (PLA; Duolink) was performed according to the manufacturer’s instructions (Sigma) using antibodies against BRD4 (Bethyl, A700-004), MITF (Santa Cruz, sc-515925), and Flag (Sigma, F1804) overnight at 4°C. Images were acquired by confocal spinning disk microscopy with a 100× or 63× objective. Each frame represents maximum intensity projection for Z-stacks captured and four to six frames were captured for each sample. The PLA units were calculated per cell as the ratio between the number of dots within the nucleus and the nucleus area (stained with DAPI, using Duolink mounting media). Each nucleus is then represented as a point in the quantification graph.

### PLA image processing and data analysis

For the automated counting of PLA emitters per nucleus in batch mode, an ImageJ (Fiji) macro was implemented [46]. In each image, the nuclei were first segmented by applying a Gaussian blur and using standard thresholding algorithms, such as Otsu. The segmented nuclei were then converted into ImageJ ROIs using the MorpholibJ [47] and PTBIOP (https://www.epfl.ch/research/facilities/ptbiop/image-processing/) plugins. For each ROI (nucleus), the number of PLA emitters was determined by identifying local maxima following blob detection via the difference of Gaussian (DoG) algorithm.

### Quantification and statistical analyses

Statistical analyses for all assays were performed with GraphPad Prism software, using one-way or two-way analysis of variance (ANOVA), or Student’s *t*-test.

## Results

### SETD6 modulates gene expression and cellular properties in melanoma cells

**Figure 1.**
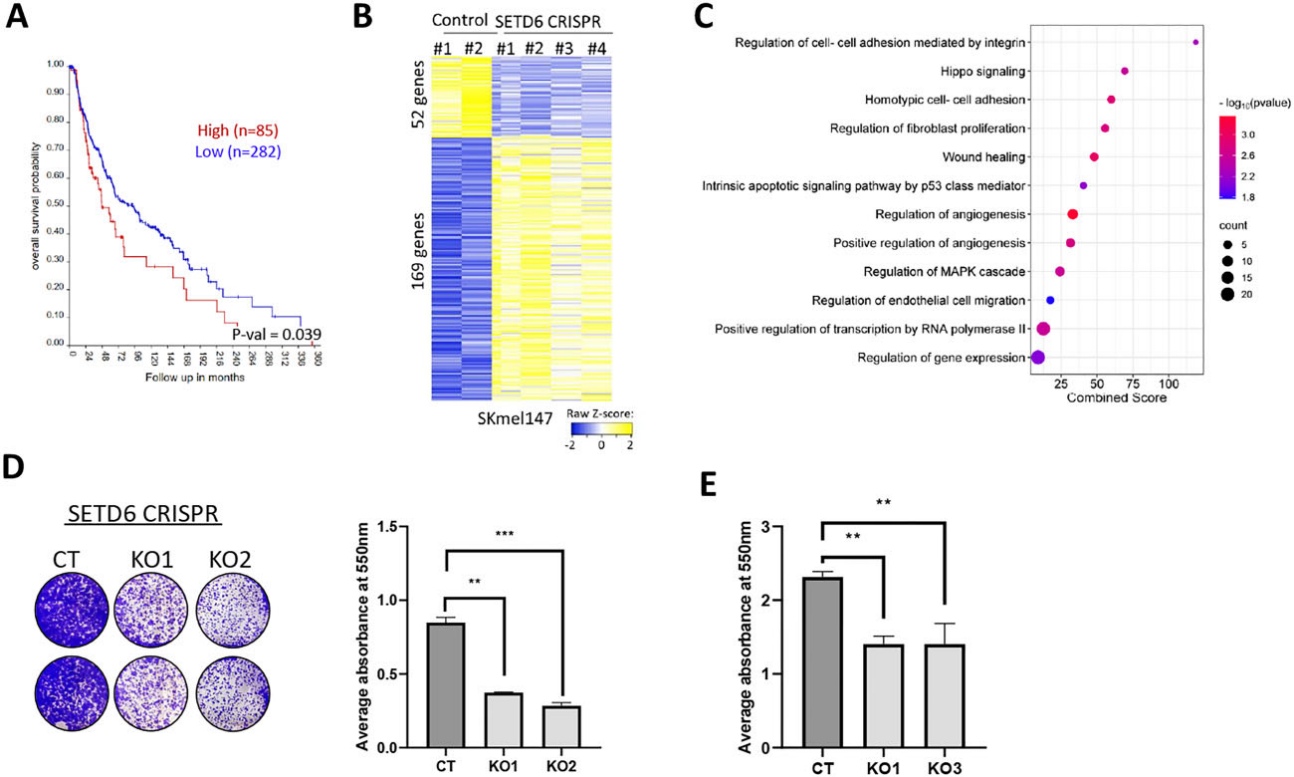
SETD6 regulates gene expression and cellular properties of melanoma cells. (**A**) Kaplan–Meier survival curve for high versus low levels of SETD6 in melanoma patients (TCGA database, *P*= .039), created using R2 genomics analysis and visualization platform (https://hgserver1.amc.nl/). (**B**) Heatmap showing up- and downregulated genes from RNA-sequencing analysis of two SETD6 control and four KO SKmel147 cells independent clones. Yellow and blue colors represent high and low expression levels, respectively. (**C**) Selected pathways from GO analysis of the DEGs were analyzed. Circle size represents the count of DEGs related to each pathway. (**D**) Colony-formation assay for SETD6 CT and KO SKmel147 cells. Images of colonies stained with crystal violet and crystal violet-stained cells were dissolved in 2% SDS and the absorbance at 550 nm was measured, whereby error bars represent the SEM. Statistical analysis was performed for two experimental repeats using one-way ANOVA (**P< .01). (**E**) Adhesion assay for SKmel147 parental, SETD6 CRISPR CT, and KO cells. Adherent cells stained with crystal violet, dissolved, and analyzed as described in panel (D).

Kaplan–Meier survival analysis of 367 melanoma patients with low (*n* = 282) or high (*n* = 85) expression of SETD6 revealed that a high level of SETD6 correlates with poor patient prognosis (Fig. 1A). These findings suggest that SETD6 may have a functional role in melanoma pathobiology. To test SETD6’s role in transcription regulation in melanoma, we performed RNA-sequencing experiments for control (CT) and KO of SETD6 in SKmel147 melanoma cells (four independent gRNA clones), which we have generated and validated by sequencing (Supplementary Fig. S1). Fifty-two downregulated genes and 169 upregulated genes were observed upon depletion of SETD6 (Fig. 1B). GO analysis revealed that the differential expressed genes are enriched in pathways such as cell proliferation and cell adhesion, two processes that are linked to melanoma as well as SETD6 enzymatic activity [48–50] (Fig. 1C). Consistent with these observations, KO of SETD6 in SKmel147 cells attenuates colony formation compared to the control cells (Fig. 1D). In a cell adhesion assay, we observed a significant decrease in the adhesive properties of SETD6-depleted cells compared to the control cells (Fig. 1E). Taken together, these results indicate that SETD6 influences gene expression profiles in melanoma cells, thereby promoting colony formation and cell adhesion.

### SETD6 interacts with and methylates BRD4 in melanoma cells

**Figure 2.**
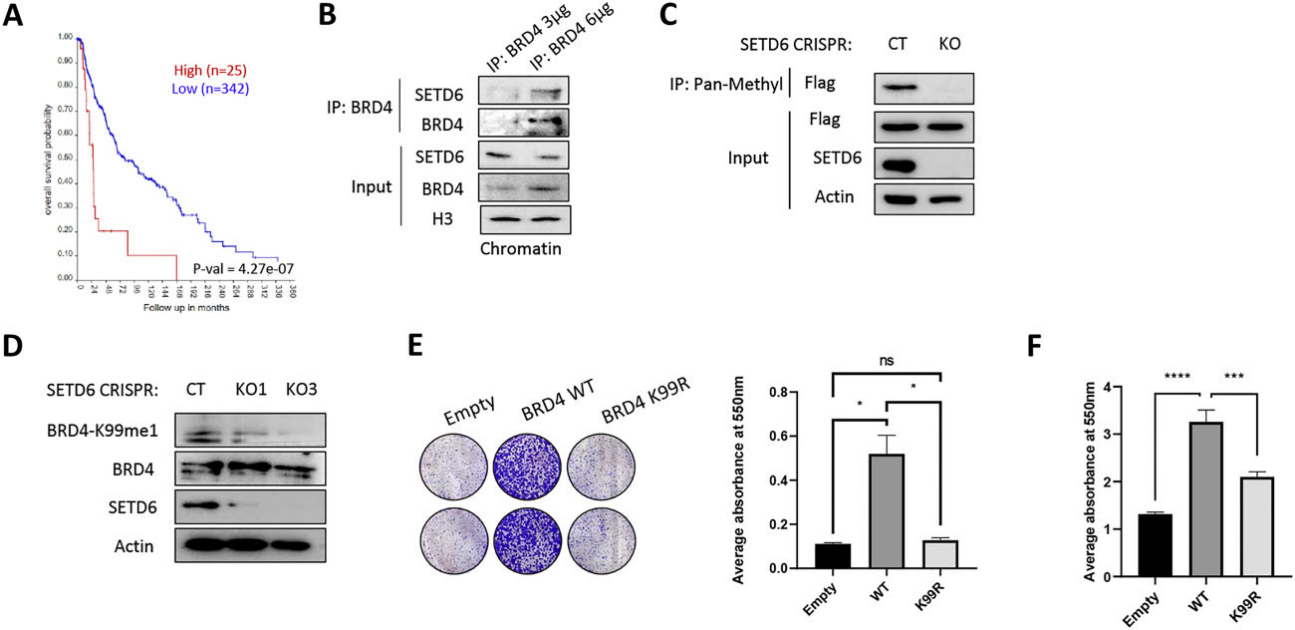
SETD6 interacts with BRD4 and methylates it in melanoma cells. (**A**) Kaplan–Meier survival curve for high versus low levels of BRD4 in melanoma patients (TCGA database, *P*= 4.27e−07) created using R2 genomics analysis and visualization platform. (**B**) Chromatin extract from SKmel147 cells were immunoprecipitated with anti-BRD4 antibody, followed by WB with the indicated antibodies. Input: levels of BRD4, SETD6, and histone-3 (loading control) in the total chromatin extracts. (**C**) Cellular methylation assay whereby SETD6 CT and KO cells were transfected with Flag BRD4 WT or Flag BRD4 K99R plasmids. Cell lysates were immunoprecipitated with preconjugated pan-methyl A/G agarose beads, and proteins in the immunoprecipitate and input samples were detected by WB with indicated antibodies. (**D**) WB analysis for CT and two SETD6 KO cells (KO1 and KO3) with the indicated antibodies. BRD4-K99me1 (U292-FT): an antibody that specifically recognizes methylated BRD4 at K99. (**E**) Colony-formation assay for SKmel147 cells stably expressing empty, Flag-BRD4 WT, and Flag-BRD4 K99R. Error bars represent the SEM. Statistical analysis was performed for two experimental repeats using one-way ANOVA (ns = not significant, *P< .05). (**F**) Adhesion assay for SKmel147 cells stably expressing empty, Flag-BRD4 WT, and Flag-BRD4 K99R. Adherent cells stained with crystal violet, dissolved, and analyzed as described in panel (F) (***P< .001, ****P< .0001).

In a recent study from our lab, we found that SETD6 methylates chromatin-bound BRD4 on lysine-99 (K99). This methylation influences gene expression by selectively recruiting the TF E2F1 to its target genes in breast cancer cells [28]. Kaplan–Meier survival analysis of 367 melanoma patients with low (*n* = 342) or high (*n* = 25) expression of BRD4 revealed that a high level of BRD4 expression is correlated with poor patient prognosis (Fig. 2A). Interestingly, this is in agreement with the same trend observed for SETD6 (Fig. 1A). These findings raised the possibility that SETD6 methylation of BRD4 at K99 might occur in melanoma cells. To address this hypothesis, we first immunoprecipitated endogenous BRD4 in melanoma cells and confirmed its interaction with endogenous SETD6 at chromatin (Fig. 2B). Next, to test whether BRD4 is a substrate for methylation by SETD6 in melanoma, we used a lysine pan-methyl antibody in control and SETD6 KO cells. As shown in Fig. 2C, our results suggest that overexpressed Flag-BRD4 is methylated in a SETD6-dependent manner. To test whether BRD4 is methylated at K99, we probed cell lysate derived from SETD6 control and KO cells with BRD4 K99me1 antibody [28] (Fig. 2D). We observed that the level of BRD4K99me1 was reduced in the SETD6 KO cells. The residual signal observed in the SETD6-depleted cells (Fig. 2D, lanes 2 and 3) comes from the endogenous BRD4 protein, which is still present. To validate this observation, we utilized a lysine pan-methyl antibody in both Flag-BRD4 WT (Wild-Type) and Flag-BRD4 K99R mutant overexpressed cells. The results revealed a reduction in the methylation of BRD4 K99R mutant compared to WT (Supplementary Fig. S2A). Phenotypically, colony formation assay was performed to test melanoma cell proliferation using SKmel147 stably expressing empty vector, BRD4 WT, or BRD4 K99R mutant, which cannot be methylated by SETD6 (Fig. 2E and Supplementary Fig. S2B for expression validation). SKmel147 cells expressing BRD4 WT demonstrate augmented colony formation compared to the empty vector control. Consistent with the experiments shown in Fig. 1D where SETD6 was depleted, cells expressing BRD4 K99R formed fewer colonies compared to BRD4 WT cells. We also observed a significant decrease in the adhesion ability of BRD4 K99R cells compared to the BRD4 WT-expressing cells (Fig. 2F). Altogether, these findings suggest that SETD6 binds and methylates BRD4 at K99 in melanoma cells to positively regulate cell proliferation and adhesion.

### BRD4 K99 methylation by SETD6 regulates its genomic occupancy

**Figure 3.**
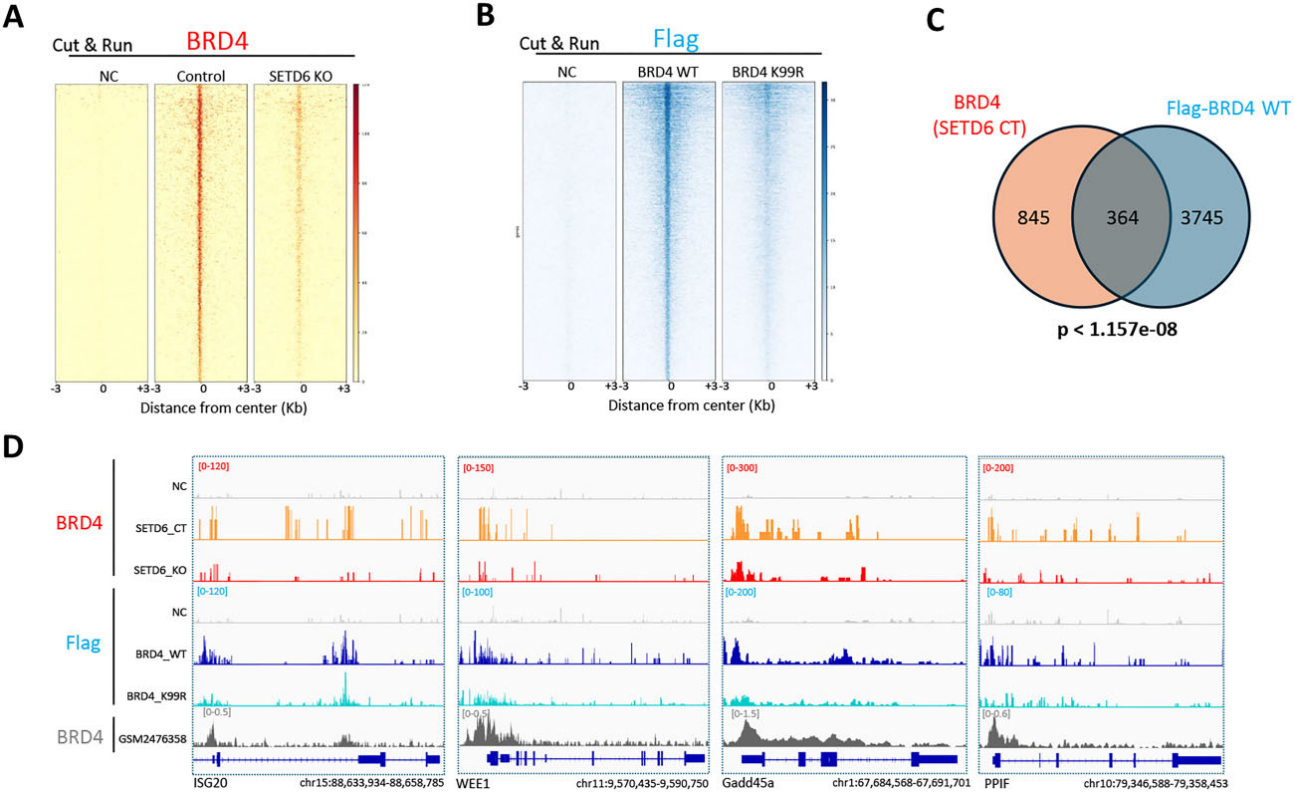
BRD4 K99 methylation by SETD6 affects its genomic distribution. (**A**) Heatmaps showing CUT&RUN read densities for IgG (NC) and BRD4 across BRD4 genomic peaks in SETD6 control and KO SKmel147 cells. (**B**) Heatmaps showing CUT&RUN read densities for IgG (NC) and Flag-BRD4 across Flag-BRD4 genomic peaks in SKmel147 cells expressing Flag-BRD4 WT or Flag-BRD4 K99R. (**C**) Venn diagram showing common genes for BRD4 in SETD6 CT cells and Flag in BRD4 WT cells as identified in the CUT&RUN analyses. (**D**) Representative binding profiles of four genomic regions.

Given that BRD4 is a transcription regulator [6], we examined whether SETD6 regulates endogenous BRD4 genomic occupancy. To do so, we performed CUT&RUN assays [51] in SKmel147 cell control and SETD6 KO cells (Fig. 3A). We found a significant decrease of BRD4 enrichment in SETD6 KO cells, which may suggest that this effect is mediated by BRD4 methylation at K99. To test this hypothesis, we performed a CUT&RUN using Flag antibody for cells stably expressing Flag-BRD4 WT or K99R mutant (Fig. 3B). Consistently, we found a significant decrease of enrichment in BRD4 K99R cells compared to BRD4 WT. To determine the genomic locations that are SETD6 and BRD4 K99me dependent, we intersected the annotated peaks of endogenous BRD4 in SETD6 control cells (Fig. 3A) with overexpressed Flag-BRD4 WT cells (Fig. 3B). The analysis revealed 364 significant shared loci (Fig. 3C). Genome browser view of the CUT&RUN experiments is presented in Fig. 3D. ChIP-seq tracks for BRD4 (GSM2476358) were added for comparison. These results demonstrate that BRD4 methylation at K99 by SETD6 affects its genomic distribution and is linked to the regulation of oncogenic related pathways.

### SETD6 regulates MITF genomic distribution

**Figure 4.**
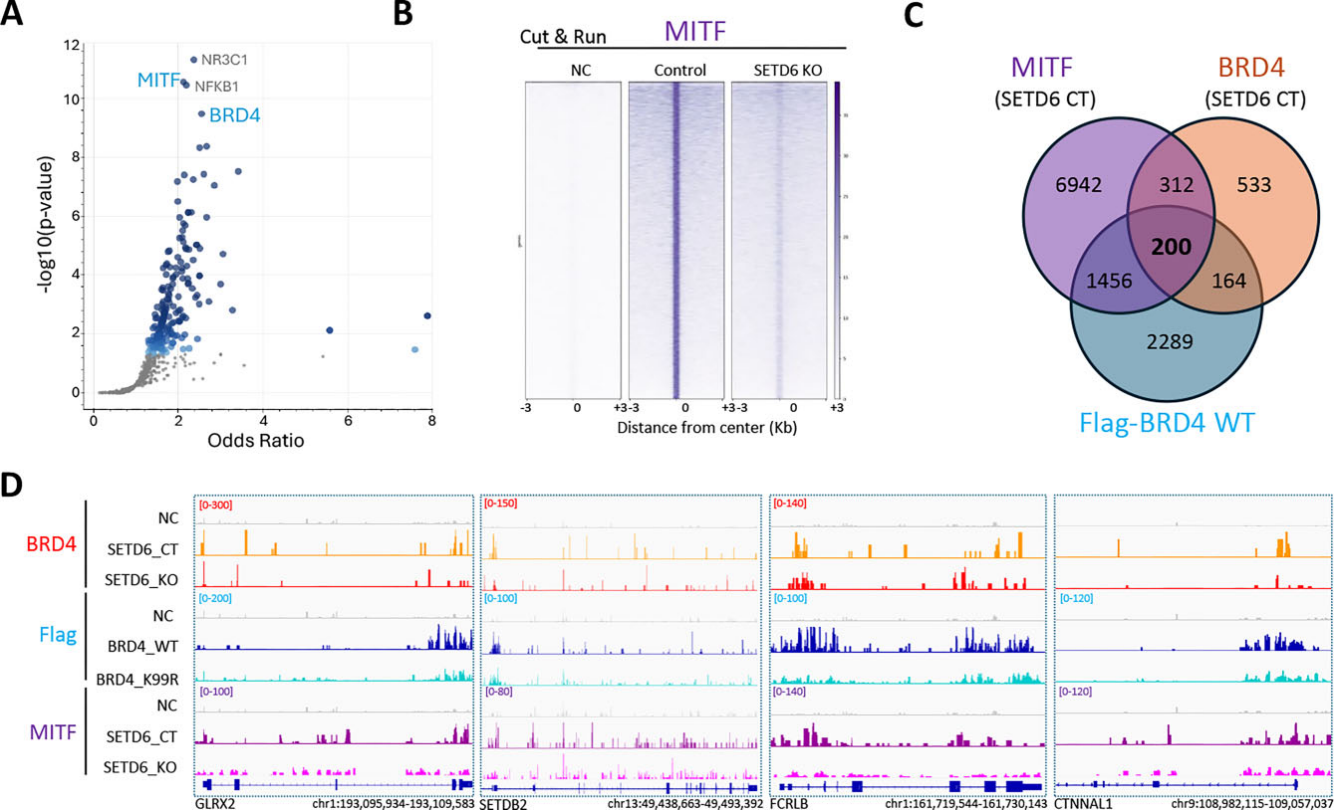
SETD6 interacts with MITF and regulates its genomic distribution. (**A**) ChEA for the 364 shared peaks presented in Fig. 3C . Blue points represent a significant TF. Smaller gray points represent nonsignificant terms. (**B**) Heatmaps showing CUT&RUN read densities for IgG (NC) and MITF across MITF genomic peaks in SETD6 control and KO SKmel147 cells. (**C**) Venn diagram showing common genes for BRD4, MITF in SETD6 CT cells and Flag-BRD4 in Flag-BRD4 WT cells as identified in the CUT&RUN analysis. (**D**) Representative binding profiles of three genomic regions.

ChEA [52] analysis on the 364 shared loci (Fig. 3C) revealed significant enrichment of several TF (Fig. 4A). As expected, BRD4 was among the four most significant ones. Both NFKB1 and NR3C1 were shown before to be associated with BRD4 cellular activity [53–55]. We were particularly interested in MITF, which is a crucial regulator of melanocyte development, controlling differentiation, cell-cycle progression, pigmentation, and melanocyte survival [1]. It is also identified as an amplified oncogene in some human melanomas [1]. Interestingly, a direct interaction between recombinant SETD6 and MITF was observed using an enzyme-linked immunosorbent assay (Supplementary Fig. S3A), RelA served as positive control for the experiment [24]. The physical interaction between SETD6 and MITF was also validated in cells through an IP experiment at the chromatin level (Supplementary Fig. S3B). These findings raised the hypothesis that SETD6 might also regulate MITF genomic distribution and activity in melanoma cells. To address this hypothesis, we performed a CUT&RUN experiment for endogenous MITF in CT and SETD6 KO cells (Fig. 4B). The results suggest that the KO of SETD6 reduces MITF recruitment or retention to its genomic binding sites. Intersection of the annotated peaks for BRD4, Flag-BRD4, and MITF revealed 200 shared genes (Fig. 4C). A snapshot for several peaks is presented in Fig. 4D.

KEGG analysis for these shared genes revealed significant enrichment for genes involved in cell adhesion (Supplementary Fig. S4). These findings may suggest a functional interplay between BRD4, MITF, and SETD6.

### The physical interaction between BRD4 and MITF is mediated by SETD6

**Figure 5.**
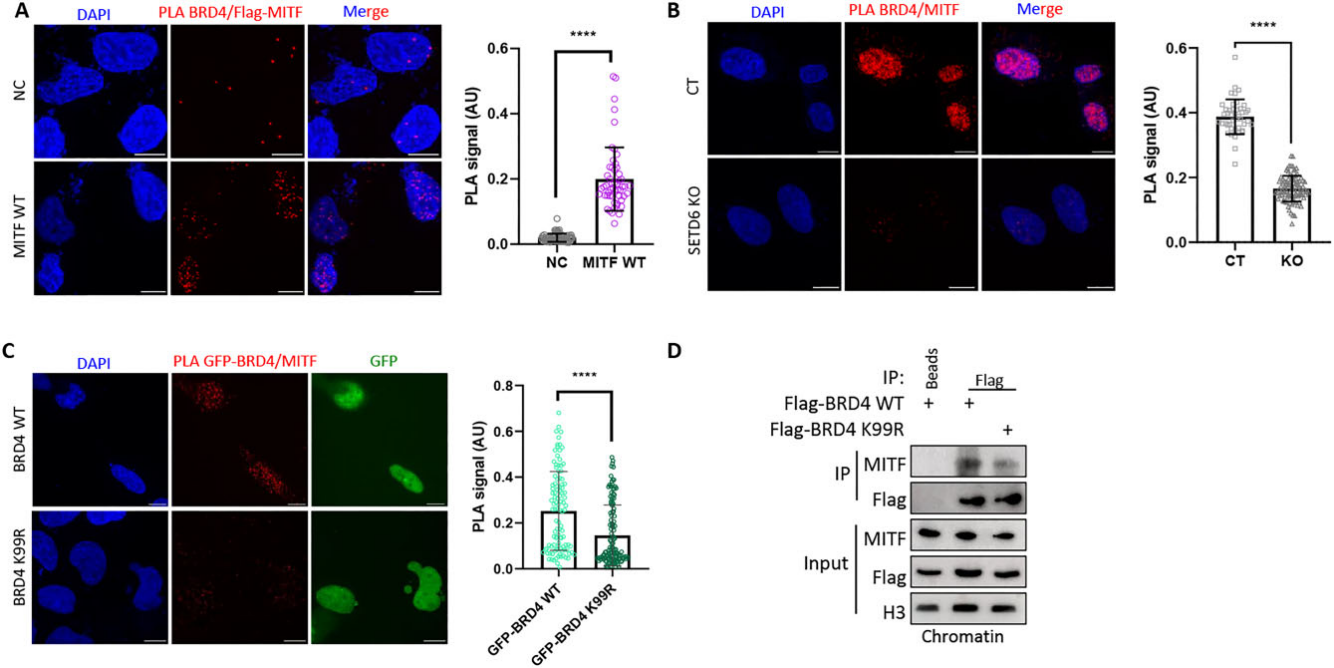
BRD4 interacts with MITF in a SETD6-dependant manner. (**A**) Left: Representative images and signal quantification (PLA dots per nucleus, AU) of PLA detecting BRD4 and Flag-MITF proximity in SKmel147 cells compared to negative control (NC) with no primary BRD4 antibody. Red dots represent PLA signal for MITF–BRD4 proximity. Scale bar = 10 µm. Right: Quantification of PLA signal per sample. Statistical analysis was performed using Student’s *t*-test (****P< .0001). (**B**) Left: Representative images of PLA detecting BRD4 and MITF proximity in SKmel147 SETD6 CT and KO cells. Red dots represent PLA signal for MITF–BRD4 proximity. Scale bar = 10 µm. Right: PLA signal quantification (PLA dots per nucleus, AU) for each sample. Statistical analysis was performed using Student’s *t*-test (****P< .0001). (**C**) Left: Representative images and signal quantification (PLA dots per nucleus, AU) of PLA detecting GFP-BRD4 and MITF in SKmel147 cells overexpressing GFP-BRD4 WT and GFP-BRD4 K99R. Red dots represent PLA signal for MITF–BRD4 proximity. Scale bar = 10 µm. Right: Quantification of PLA signal per sample. Statistical analysis was performed using Student’s *t*-test (****P< .0001). (**D**) Chromatin extract from SKmel147 cells overexpressing Flag-BRD4 WT or K99R were immunoprecipitated with anti-Flag antibody, followed by WB with the indicated antibodies. Input: levels of MITF, Flag, and histone-3 (loading control) in the total chromatin extracts.

To address this hypothesis, we decided to check whether BRD4 interacts with MITF. We confirmed that the physical interaction between endogenous BRD4 and overexpressed Flag-MITF occurs in cells using a PLA (Fig. 5A). Using a complementary approach, we found that endogenous MITF co-immunoprecipitated with endogenous chromatin-bound BRD4 (Supplementary Fig. S5). We hypothesized that the physical interaction between the proteins depends on the presence of SETD6. To address this possibility, we performed a PLA experiment using specific antibodies to endogenous BRD4 and MITF in SETD6 control and KO cells (Fig. 5B). These experiments revealed that SETD6 KO abolishes the interaction between BRD4 and MITF, suggesting that the interaction between these proteins in cells is SETD6 dependent. Given that SETD6 promotes BRD4-K99me1, we hypothesized that this specific methylation event may regulate the interaction between BRD4 and MITF. Therefore, we monitored the interaction of endogenous MITF with BRD4 WT or BRD4 K99R mutant using PLA (Fig. 5C). As expected, we observed a significant reduction of PLA signal in BRD4 K99R cells compared to the BRD4 WT cells, indicating that the methylation of BRD4 at K99 positively regulates its physical interaction with MITF. To support these findings, we performed an IP using either BRD4 WT or K99R mutant and observed a reduced interaction between BRD4 and MITF in the mutant (Fig. 5D). Taking together, these results suggest that the physical interaction between BRD4 and MITF is SETD6 and BRD4-K99 methylation dependent.

### The bromodomain of BRD4 binds to acetylated MITF

**Figure 6.**
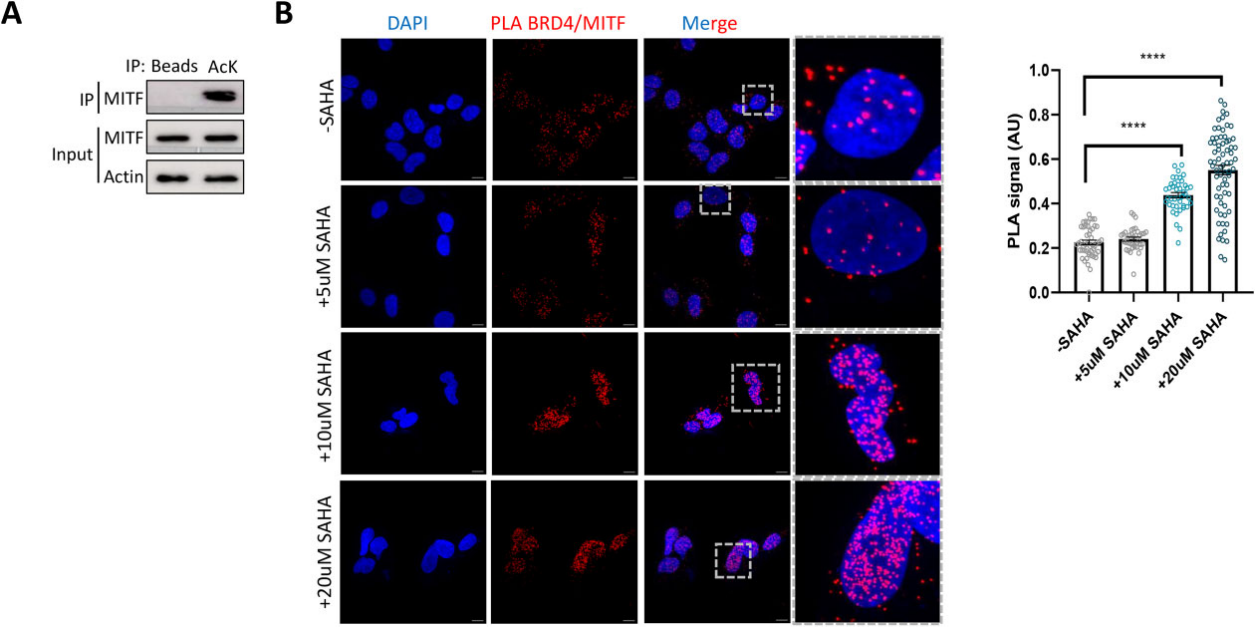
Acetylation of MITF increases its interaction with BRD4. (**A**) Acetylation assay in cells. SKmel147 cell lysates were subjected to IP with preconjugated pan-acetyl lysine A/G agarose beads. Proteins in the immunoprecipitate and input samples were detected by WB with the indicated antibodies. (**B**) Representative images and signal quantification (PLA dots per nucleus, AU) of PLA detecting BRD4 and MITF proximity in SKmel147 cells that were untreated or treated with SAHA for 4 h. Red dots represent a PLA signal for MITF–BRD4 proximity. Scale bar = 10 µm. Right: Quantification of PLA signal per sample. Statistical analysis was performed using Student’s *t*-test (****P< .0001).

**Figure 7.**
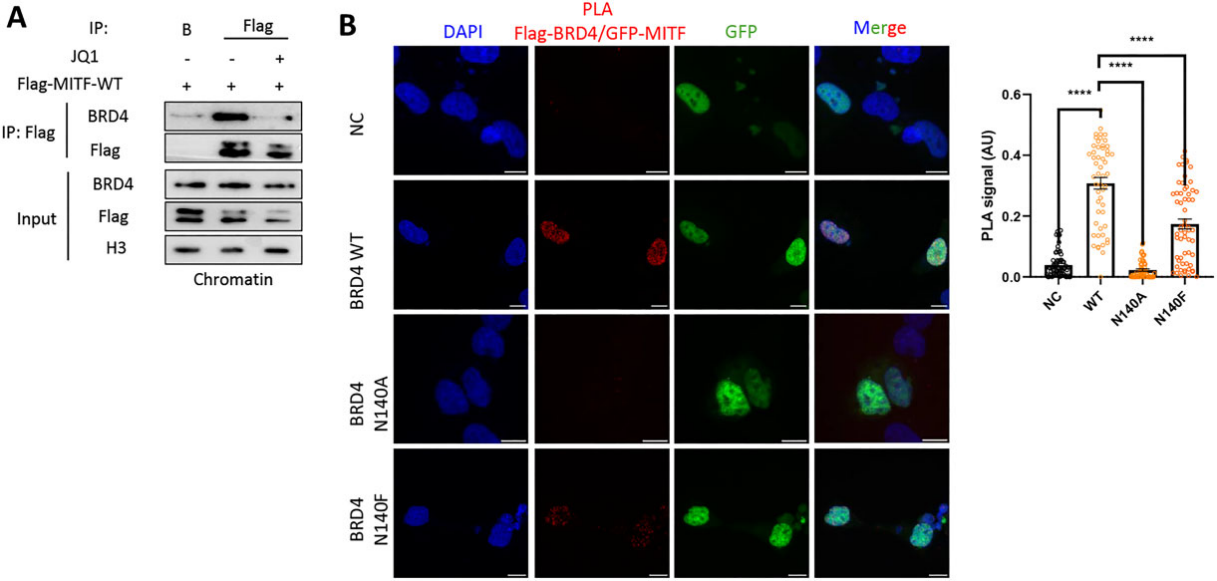
The BD of BRD4 binds to acetylated MITF. (**A**) SKmel147 cells were transfected with FLAG-MITF-WT and treated with 1 μM JQ1, where indicated. Chromatin fractions were then immunoprecipitated (IP) with a Flag antibody followed by WB with the indicated antibodies. Beads (B) served as negative control for the IP. (**B**) Representative images and signal quantification (PLA dots per nucleus, AU) of PLA to detect the proximity of Flag-BRD4 and MITF in SKmel147 cells overexpressing Flag-BRD4 WT, Flag-BRD4 N140A, or Flag-BRD4 N140F mutants. Red dots represent a PLA signal for MITF–BRD4 proximity. Scale bar = 10 µm. Right: Quantification of PLA signal per sample. Statistical analysis was performed using Student’s *t*-test (****P< .0001).

Since MITF is known to be acetylated on several lysine residues [56, 57], we hypothesized that the BRD4–MITF interaction is mediated by the BD of BRD4 [6, 7] and acetylated MITF. The usage of a pan-acetyl antibody in IP experiments allowed us to determine that MITF is indeed acetylated in SKmel147 cells (Fig. 6A). To test whether the interaction with BRD4 is mediated by the acetylation of MITF, we performed a PLA experiment in the presence of increasing concentrations of SAHA, a potent inhibitor of histone deacetylases [58]. As shown in Fig. 6B, we observed a stronger interaction between BRD4 and MITF in a SAHA dose-dependent manner. Next, we performed an IP experiment in the absence or presence of the BD inhibitor, JQ1 [59], to inhibit BRD4 ability to bind its acetylated partners. The results demonstrate that the interaction between overexpressed Flag-MITF and endogenous BRD4 decreased in the presence of JQ1 (Fig. 7A). In a reciprocal experiment, the interaction between overexpressed GFP-BRD4 and endogenous MITF decreased in the presence of JQ1 (Supplementary Fig. S6). These experiments indicate that the BD of BRD4 mediates the interaction with MITF.

**Figure 8.**
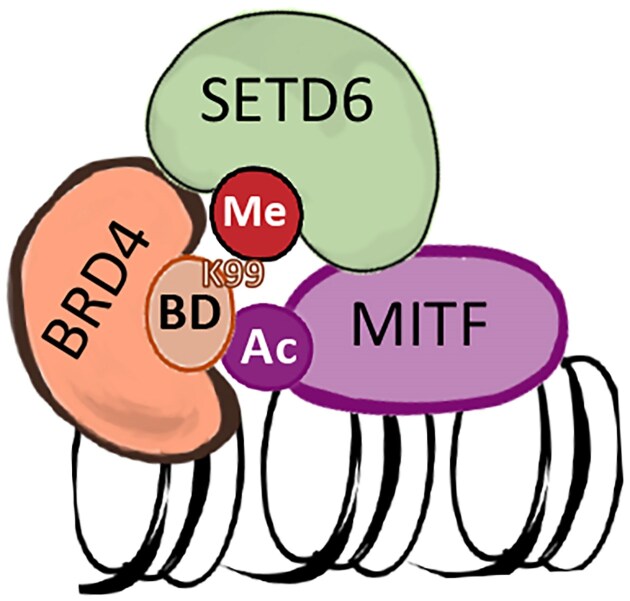
BRD4 interacts with acetylated MITF by its BD in a SETD6-dependent manner. Schematic model illustrating the proposed SETD6–BRD4–MITF axis.

The N140 residue of BRD4 is located within its first BD and mutations at this position have previously been shown to impair BRD4 function. Notably, the well-characterized inhibitor JQ1 has been reported to form a direct hydrogen bond with N140, highlighting its importance in ligand binding. Both N140A and N140F mutants seem to reduce BRD4 affinity for acetylated lysine [60]. To test the effect of these mutants on BRD4 ability to interact with MITF, we performed PLA experiment (Fig. 7B). We observed a significant reduction of PLA signal in both BRD4 N140A and N140F cells compared to the BRD4 WT cells, which provides further evidence that the interaction between MITF and BRD4 occurs through the BRD4 BD. Taken together, our findings suggest a model by which the interaction between MITF and BRD4 is mediated by the acetylation of MITF and the BD of BRD4 in a SETD6-dependent manner (Fig. 8). We suggest that the formation of this complex at chromatin has a role in the transcription regulation of genes important for melanoma initiation and progression.

## Discussion

The PKMT SETD6 has a fundamental role in the regulation of several biological processes and pathologies including cancer [17–22, 24–30]. The tight and significant correlation between the high SETD6 expression level and poor survival of melanoma patients led us to study its role in this cancer. RNA sequencing revealed that SETD6 regulates the expression of genes that are related to various pathways in melanoma. Accordingly, alterations in cellular phenotypes, such as cell proliferation and adhesion, were seen after SETD6 KO, providing the first evidence of its involvement in melanoma. These phenotypes are also consistent with previous studies in human cancers such as breast, glioma, and others [18, 27, 28, 30]. The increasing cellular functions of SETD6 in both normal and disease states make it an attractive candidate for therapeutic use.

We have previously shown that SETD6 specifically methylates BRD4 at K99 in a breast cancer cellular model, which in turn regulates the transcription of genes that control mRNA translation [28]. Here, we provide evidence that BRD4 methylation by SETD6 at K99 plays an important role also in melanoma cells. Our biochemical and genomics analysis revealed that SETD6-mediated methylation of BRD4 at chromatin is essential to drive selective gene expression programs by recruitment of BRD4 to different genomic loci and genes. This effect is both SETD6 and BRD4 K99 methylation dependent. Mechanistically, we observed two independent modes of action in breast cancer versus melanoma cells. In breast cancer cells, BRD4 methylation specifically determines the recruitment of the TF E2F1 to selected target genes. However, here we provide evidence that in melanoma cells the methylation of BRD4 is required for the recruitment of MITF. It seems that methylated BRD4 at chromatin might serve as a scaffold for recruiting different TFs to allow selective and efficient regulation of gene expression programs.

Our genomic analysis revealed that MITF genomic occupancy is regulated by SETD6, similar to BRD4. Interestingly, our study also uncovered that SETD6 physically interacts with MITF. This observation prompted us to investigate whether there is an overlap between the target genes of BRD4 and MITF that are regulated by the presence of SETD6. Indeed, we observed common genomic loci for BRD4 and MITF that are affected by SETD6. Consistent with our findings, a recent study showed that BRD4 and MITF physically interact with each other in melanocyte cells and this interaction controls the production of melanin [2]. These observations led us to examine whether BRD4 and MITF co-occupy the same genomic regions. Our data revealed that this co-occupancy also depends on SETD6 and the methylation of BRD4 at K99. Future work will reveal whether SETD6 and the methylation of BRD4 are also required for BRD4 and MITF transcriptional activity in regulating specific gene expression programs in melanoma.

Previously, MITF acetylation by p300 was shown to direct MITF to distinct genomic locations, thereby coordinating the expression of genes that regulate melanocyte and melanoma proliferation and differentiation [56, 57]. This led us to investigate whether the newly identified BRD4–MITF interaction in melanoma cells is mediated by the BD of BRD4 and acetylated MITF. We tackled this question by either inhibiting the BD of BRD4 or inducing MITF acetylation in cells. Our findings not only support this model, but also suggest that it is regulated by SETD6 and the methylation of BRD4 at K99. A particularly intriguing direction for future research is to explore the direct interaction between MITF and SETD6. Further investigation into the specific acetylation sites in MITF facilitating this interaction, as well as the potential crosstalk with nearby PTMs on MITF, would be of significant interest as well.

In summary, we uncovered a new functional crosstalk at chromatin, between SETD6, BRD4, and MITF. The formation of this chromatin-associated complex plays a critical role in selective recruitment of these TFs to different genomic loci in melanoma cells. Thus, targeting the biochemical interactions identified in this study might represent a promising strategy for inhibiting melanoma progression. The inhibition of BRD4 methylation by SETD6 or preventing BRD4 from recognizing MITF acetylation may yield similar therapeutic outcomes.

## Supplementary Material

zcaf023_Supplemental_File

## Data Availability

RNA-seq data were deposited into the Gene Expression Omnibus database under accession number GSE298161. CUT&RUN data were deposited into the Gene Expression Omnibus database under accession number GSE298161.
