# Supplementary material for: SETD6 mediates selective interaction and genomic occupancy of BRD4 and MITF in melanoma cells
Source: NAR Cancer. 2025 Aug 7;7(3):zcaf023. doi: 10.1093/narcan/zcaf023 (PMC12342177; doi:10.1093/narcan/zcaf023)
Supplement: zcaf023_Supplemental_File [file zcaf023_supplemental_file.pdf]

Supplementary information

SETD6 Mediates Selective Interaction and Genomic Occupancy of BRD4 and MITF in Melanoma cells

Tzofit Elbaz Biton[1,2], Michal Feldman[1,2], Tomer Davidy[1,2], Nili Tickotsky Moskovitz[3], Liron Levin[3], Daniel Sevilla[4] Colin R. Goding[5], Emily Bernstein[6] and Dan Levy[1,2#]

[1]The Shraga Segal Department of Microbiology, Immunology and Genetics; [2]National Institute for Biotechnology in the Negev, Ben-Gurion University of the Negev, P.O.B. 653, Be'er-Sheva 84105, Israel; [3]Bioinformatics Core Facility, Ilse Katz Institute for Nanoscale Science and Technology, Ben-Gurion University of the Negev, Beer Sheva, Israel; [4]The Research Support Laboratories of Ilse Katz Institute for Nano-Science and Technology, Ben-Gurion University of the Negev, Beer Sheva, Israel; [5]Ludwig Institute for Cancer Research, Nuffield Department of Clinical Medicine, University of Oxford, Headington, Oxford, OX3 7DQ, UK; [6]Department of Oncological Sciences, Tisch Cancer Institute, Icahn School of Medicine at Mount Sinai, New York, New York 10029, USA

[#]Correspondence should be addressed to D.L (ledan@post.bgu.ac.il)

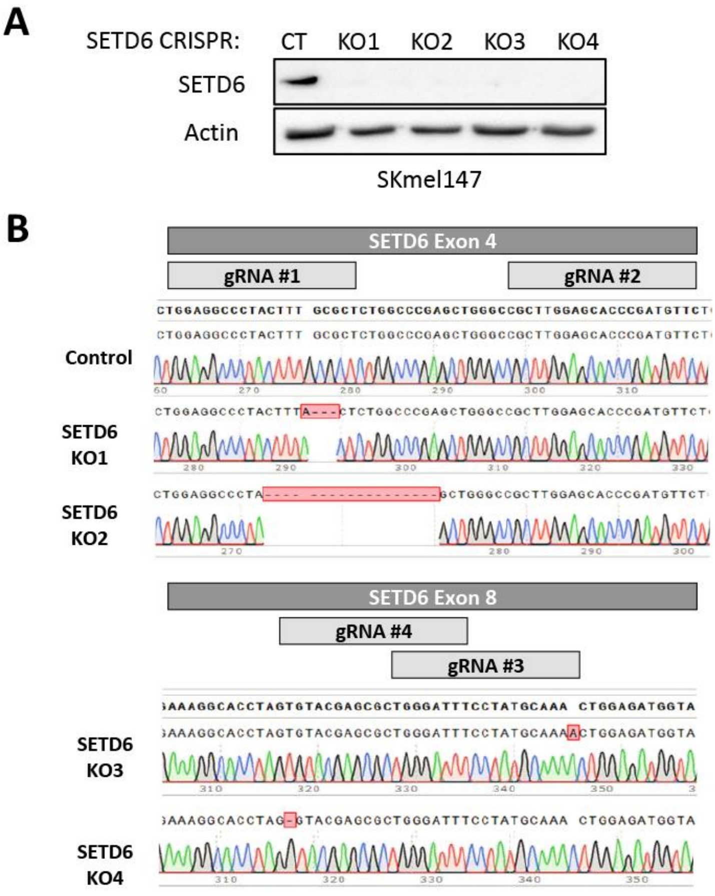

Supplementary Fig 1

Supplementary figure 1 - SETD6 CRISPR Knock-Out formation and validation by sequencing

Generation of SETD6 KO by CRISPR in SKmel147 cells. **(A)** Protein expression levels of SETD6 and Actin (loading control) were measured in SKmel147 cells by Western blot (WB). CT stands for the lenti-CRISPR pLKO_TRC005 Cas9, the numbers represent different gRNAs targeting different exons of the SETD6 gene. **(B)** Validation of CRISPR SETD6 presented by chromatograms of Sanger sequencing.

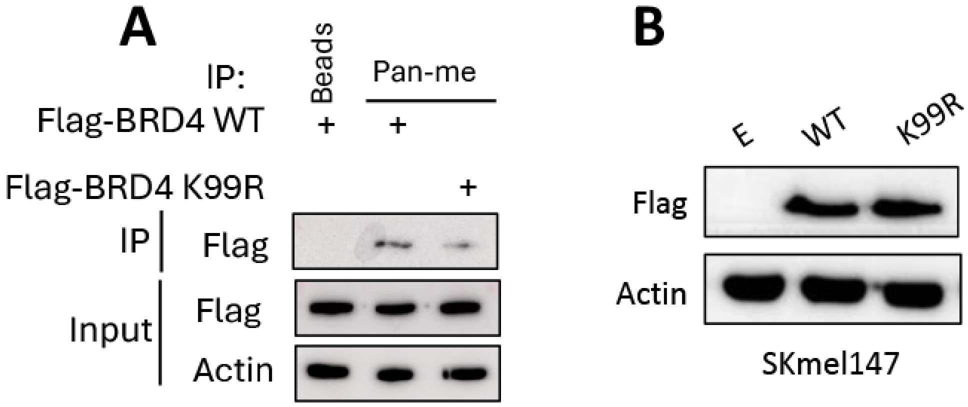

Supplementary Fig 2

Supplementary figure 2 – BRD4-K99 methylation in SKmel147 cells **(A)** SKmel147 cells were transfected with Flag BRD4 WT or Flag BRD4 K99R plasmids. Cell lysates were immunoprecipitated with pre-conjugated pan-methyl A/G agarose beads, followed by Western blot with the indicated antibodies. **(B)** WB analysis with the indicated antibodies for SKmel147 cells stably expressing Empty, BRD4 WT or BRD4 K99R plasmids.

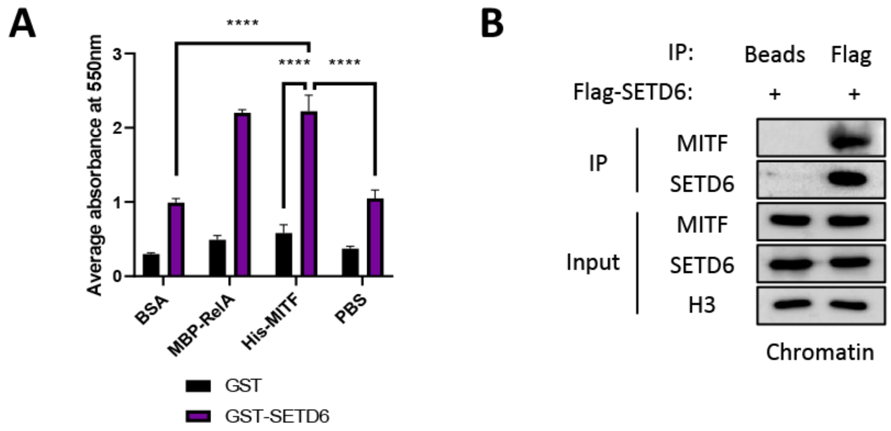

Supplementary Fig 3

Supplementary figure 3 – Physical interaction between SETD6 and MITF

(A) Interaction assay by ELISA. The 96-well plate was coated with MBP-RelA, His-MITF or BSA as negative control, and covered with GST-SETD6 or GST. The reactions were probed with primary anti-GST antibody and HRP-conjugated secondary antibody. The signal was detected at 450nm. Graph represents relative absorbance of each condition and error bars represent the SEM. Statistical analysis was performed for three experimental repeats using one-way ANOVA (**** $p<0.0001$). **(B)** Chromatin extract from SKmel147 cells overexpressed Flag-SETD6 were immunoprecipitated using A/G Flag magnetic beads and blotted with SETD6 and MITF antibodies. Input - levels of SETD6, MITF, and H3 (loading control) in the total chromatin extracts.

KEGG analysis (200 shared genes)

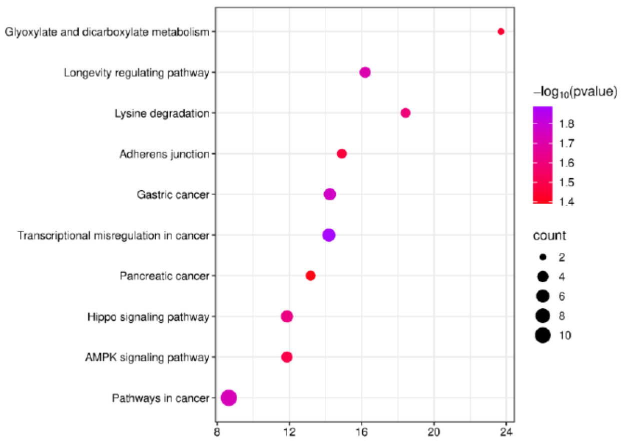

Supplementary Fig 4

Supplementary figure 4

Selected pathways from KEGG analysis of significantly 200 common genes presented in Fig. 4C.

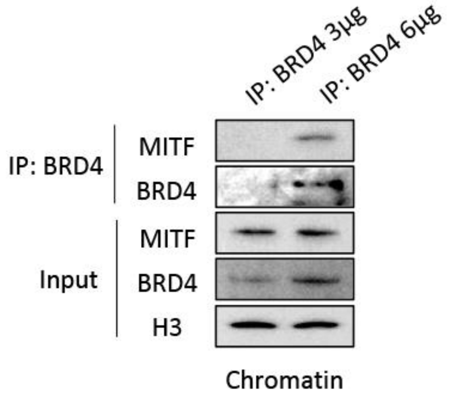

Supplementary Fig 5

Supplementary figure 5 – BRD4 interacts with MITF in cells

SKmel147 melanoma cells were IP using A/G magnetic beads that were conjugated to anti-BRD4 antibody followed by WB analysis with the indicated antibodies. Input - levels of BRD4, MITF, and Histone-3 (loading control) in the total chromatin extracts.

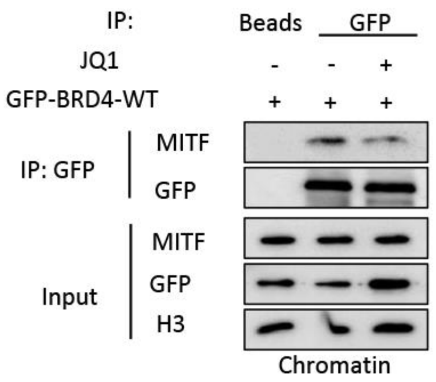

Supplementary Fig 6

Supplementary figure 6 – The bromodomain of BRD4 is required for the interaction with MITF. SKmel147 cells were transfected with GFP-BRD4-WT and treated with 1μM JQ1 where indicated. Chromatin fraction was then IP with a GFP antibody followed by WB with the indicated antibodies.

Name	Sequence (5’ to 3’)
MITF-A fw	TTAGGCGCGCCCAGTCCGAATCGGGGATCG
MITF-A rev	GGCTTAATTAACTAACAAGTGTGCTCCGTCTCTTC
SETD6 gRNA #1	TAAGGCGCGCCGGTGAAACTCTGGGAGATTCTCC
SETD6 gRNA #2	GGCTTAATTAACTAGTACAAGTCCTTGTAGATCTCCTGC
SETD6 gRNA #3	GAACAATCAGATTGCGCCTTATCTATGACAGATGTGATCTTAACTGTCGG
SETD6 gRNA #4	TCTGTCATAGATAAGGCGCAATCTGATTGTTCTCCGGAAGAAACC
BRD4 N140A fw	ATTGTTACATCTACGCCAAGCCTGGAGATGACATAGTCTTAATG
BRD4 N140A rev	TCATCTCCAGGCTTGGCGTAGATGTAACAATTTGTAAACATAGTGTTGAAGTC
BRD4 N140F fw	ATTGTTACATCTACTTCAAGCCTGGAGATGACATAGTCTTAATG
BRD4 N140F rev	TCATCTCCAGGCTTGAAGTAGATGTAACAATTTGTAAACATAGTGTTGAAGTC

Supplementary Table 1

Supplementary table 1 – Primers sequences used for cloning
